# Fluoromethylcyclopropylamine derivatives as potential *in vivo* toxicophores – A cautionary disclosure

**DOI:** 10.1016/j.bmcl.2018.12.066

**Published:** 2019-02-15

**Authors:** Ben Acton, Helen F. Small, Kate M. Smith, Alison McGonagle, Alexandra I.J. Stowell, Dominic I. James, Niall M. Hamilton, Nicola Hamilton, James R. Hitchin, Colin P. Hutton, Ian D. Waddell, Donald J. Ogilvie, Allan M. Jordan

**Affiliations:** Drug Discovery Unit, Cancer Research UK Manchester Institute, The University of Manchester, Alderley Park, Macclesfield SK10 4TG, UK

**Keywords:** GI, gastrointestinal, PARG, poly-ADP ribose glycohydrolase, PK, Pharmacokinetics, Ataxia, Animal welfare, Toxicophore

## Abstract

Fluorination of metabolic hotspots in a molecule is a common medicinal chemistry strategy to improve *in vivo* half-life and exposure and, generally, this strategy offers significant benefits. Here, we report the application of this strategy to a series of poly-ADP ribose glycohydrolase (PARG) inhibitors, resulting in unexpected *in vivo* toxicity which was attributed to this single-atom modification.

## Graphical abstract

**Figure:**
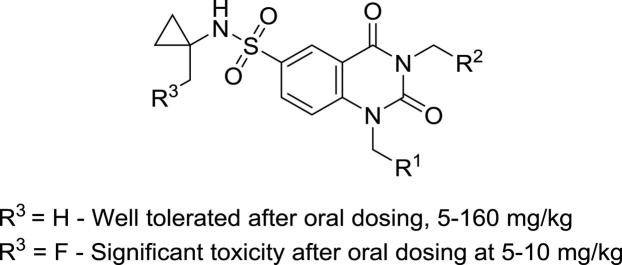

We recently described a series of quinazolinedione inhibitors of the DNA repair enzyme poly-ADP ribose glycohydrolase (PARG), typified by **1** and **2**.^1^ During the optimisation of these molecules, we discovered that these compounds suffered from a short half-life when exposed to human liver microsomes. Generally, compounds bearing two benzyl or heteroarylmethyl substituents, such as **1**, were highly potent but degraded more quickly in *in vitro* microsomal stability assays than those with a single pendant aryl moiety, such as **2**, which were around ten-fold less potent.^1^ Indeed, initial *in vivo* pharmacokinetic (PK) studies confirmed these findings and demonstrated that both molecules were unsuitable for detailed *in vivo* efficacy studies, due to their short half-lives and lack of sustained target coverage. Despite this, both **1** and **2** were well tolerated at a range of oral doses from 5 mg/kg to a maximum deliverable dose of 160 mg/kg (Fig. 1).

**Fig. 1:**
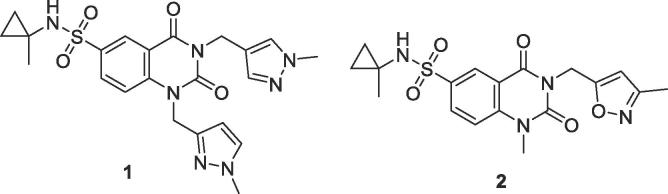
Representative quinazolinedione PARG inhibitors.

In an attempt to improve the *in vivo* PK profile, we undertook a series of metabolite identification studies, to determine the key sites of metabolism on **1** and **2**. These studies indicated the major metabolite to be the primary sulphonamides **3** and **4**, formed *via* removal of the cyclopropylmethyl moiety (Fig. 2).

**Fig. 2:**
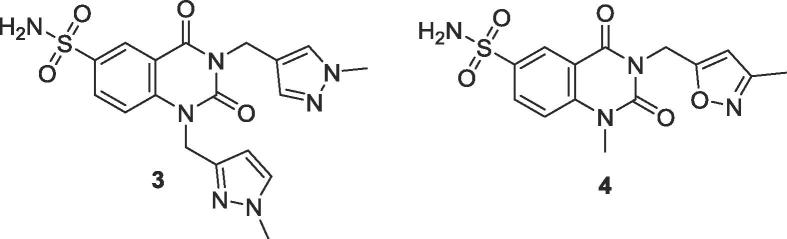
Identified primary metabolites.

We therefore undertook to replace the hydrogens of this methyl group with fluorines, a common strategy in medicinal chemistry. Whilst the di- and trifluoromethyl derivatives **6** and **7** were more stable in our human microsomal metabolism assay, both derivatives demonstrated a reduction in activity against the target enzyme (Table 1). We attributed this to the strict steric restraints in this region of the enzyme, as reported previously.^2^ However, the monofluorinated derivative **5** maintained potency and demonstrated a helpful increase in metabolic stability, particularly in human liver microsomal stability assays. In contrast, the deuterated derivative **8** failed to demonstrate any increase in metabolic stability.

**Table 1:** Improvements to metabolic stability through cyclopropyl modification.

**Figure:**
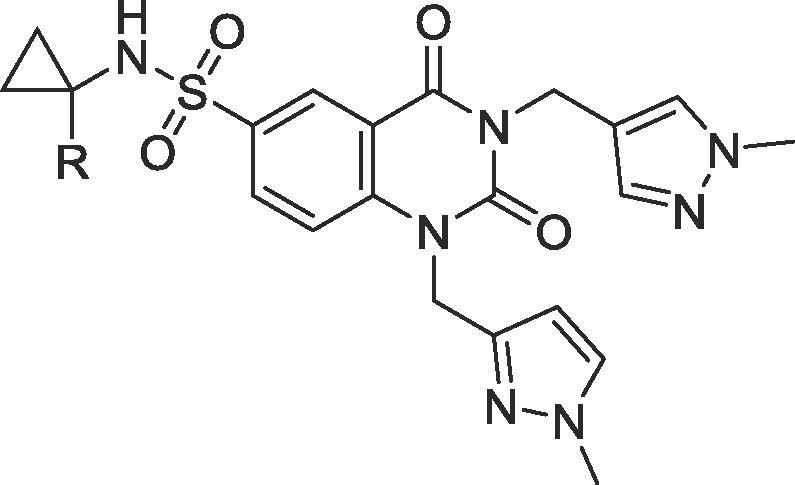

Compound	R	PARG Enzyme Activity (µM)^a^	PARG Cell Assay (µM)^a^	Mouse Microsomes T_1/2_ (minutes)	Human Microsomes T_1/2_ (minutes)
**1**	CH_3_	0.02	0.09	22	53
**5**	CH_2_F	0.02	0.17	37	82
**6**	CHF_2_	0.32	1.25	42	71
**7**	CF_3_	3.6	4.1	38	102
**8**	CD_3_	0.03	0.07	18	34

a All assays values are the geometric mean of at least two independent determinations. Detailed biochemical assay protocols^3^ and cell assay protocols^4^ have been described previously.

Given these findings, an extended series of derivatives bearing this interesting monofluorinated cyclopropyl amine were prepared and their PK properties investigated *in vivo*.^5^

Disappointingly, compound **5** (the monofluorinated derivative of the highly potent PARG inhibitor **1**) also suffered from high clearance and *in vivo*, oral bioavailability could not be adequately determined for this derivative (Table 2). In contrast, compound **10** initially showed promising activity and appeared to be well tolerated when dosed orally at 5 mg/kg. We therefore initiated a dose-escalation study to examine dose versus exposure of the compound. However, upon returning to sample at the 24 h time point in this study, one animal was found to be exhibiting reduced activity and one animal was found dead on study. Gross post-mortem examination suggested liver toxicity was the probable cause of death, with a pale yellow, mottled organ and, upon macroscopic analysis, some traces of gastrointestinal (GI) bleeding. Our following study, with derivative **12**, incorporated enhanced monitoring and here we observed the onset of ataxia shortly after the 5 h sampling time point, and exclusively in the oral dosing arm of the study, despite lower absolute and free drug exposures in these dosing arms.

**Table 2:** In vitro and in vivo parameters for key compounds.^8^

**Figure:**
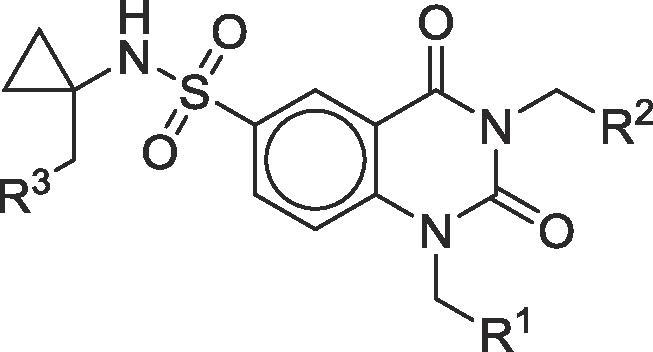

Compound	R^1^	R^2^	R^3^	PARG Enzyme Activity (µM)^a^	PARG Cell Assay (µM)^a^	Mouse Microsomes T_1/2_ (minutes)	Oral dose	Fold exposure relative to cell IC_50_^b^	Toxicity Observed
**1**			H	0.02	0.09	22	5	0.96	No
							160	350	No
**5**			F	0.02	0.17	37	5	0.18	No
**9**	CH_3_		H	0.17	0.08	47	5	6.4	No
**10**			F	0.10	0.08	102	5	15	No
							10	Not determined due to toxicity	Liver toxicity
**11**	H		H	0.39	0.33	82	5	33	No
**12**			F	0.63	0.29	285	5	61	Ataxia
**13**	CH_3_		F	1.52	6.6	121	5	0.28	Ataxia

a All assays values are the geometric mean of at least two independent determinations. Detailed biochemical assay protocols^3^ and cell assay protocols^4^ have been described previously.

b Defined as [C_max(free)_]/Cell IC_50_.

Having previously evaluated twenty four related, but non-fluorinated compounds *in vivo*, at doses ranging from 5 to 160 mg/kg and having observed no prior signs of toxicity, we felt it was unlikely that the observed idiosyncratic toxicity was arising from on-target pharmacology due to PARG inhibition, particularly as the total exposure and Cmax observed with **12** was over four-fold lower than that observed for the significantly more potent PARG inhibitor **1**. To further test this hypothesis, we decided to study compound **13**. This derivative was closely related to **10** and **12**, but was significantly less potent when tested in our PARG biochemical and cellular assays. Once again, this compound was found to induce ataxia just after the 5 h time point, suggesting the toxicity does not arise from on-target inhibition of PARG.

We have now evaluated eleven compounds bearing the fluoromethylcyclopropyl amine moiety and, with the exception of **5**, which was very rapidly cleared after oral dosing, all derivatives have induced the onset of ataxia after oral dosing at around the five hour post-dose time point. Despite extensive investigation, we found no correlation with Cmax, AUC, absolute PARG inhibitory activity or exposure of free drug above PARG cellular IC_50_ or IC_90_. Indeed, the only common factor across all these compounds appears to be the presence of the monofluorinated amine.

Given the resurgence of interest in the use of cyclopropyl derivatives in medicinal chemistry,^6^ and the extensive use of fluorination of metabolic “soft spots” in order to improve metabolic profile,^7^ it is perhaps unsurprising that we have brought these two moieties together in our molecule. However, the off-target effects we have observed with this combination were unexpected and cause for significant concern. Whilst we have no specific evidence to date, we suggest that the observance of toxicity in the oral dosing arm of our *in vivo* studies, and not in the intravenous dosing arm, may be related to metabolic degradation of the fluoromethylcyclopropyl derivative in the GI tract, leading to a circulating active metabolite which induces the observed idiosyncratic toxicity.

Regardless of the underlying mechanism, we believe it is important to highlight these findings and caution against the use of this fluorinated amine, in the interests of animal welfare and the reduction of suffering, in line with our commitments under the Animals (Scientific Procedures) Act 1986 for animal experiments.

## Appendix A Supplementary data

### MOL files

The following ZIP file contains the MOL files of the most important compounds referred to in this article.

MOL files ZIP file containing the MOL file of the most important compounds in this article
