# Supplementary material for: Fluoromethylcyclopropylamine derivatives as potential in vivo toxicophores – A cautionary disclosure
Source: Bioorg Med Chem Lett. 2019 Feb 15;29(4):560–2. doi: 10.1016/j.bmcl.2018.12.066 (PMC6376317; doi:10.1016/j.bmcl.2018.12.066)
Supplement: Supplementary Data 1 [file mmc1.docx]

**Fluoromethylcyclopropylamine derivatives as potential *in vivo* toxicophores – a cautionary disclosure.**

Ben Acton, Helen F. Small, Kate M. Smith, Alison McGonagle, Alexandra I.J. Stowell, Dominic I. James, Niall M. Hamilton, Nicola Hamilton, James R. Hitchin, Colin P. Hutton, Ian D. Waddell, Donald J. Ogilvie and Allan M. Jordan^*^.

*Drug Discovery Unit, Cancer Research UK Manchester Institute, The University of Manchester, Alderley Park, Macclesfield, SK10 4TG. U.K.*

**Compound synthesis**

Detailed synthetic routes to all the compounds described in this manuscript can be found in McGonagle, AE et al., WO 2016/092326.

**Analytical Data Summary for all Compounds.**

| **COMPOUND** | **pH 4** | | | | **pH 10** | | | | **NMR**  **Purity** |
| --- | --- | --- | --- | --- | --- | --- | --- | --- | --- |
|  | **RT^a^** | **Obs MW^b^** | **Adduct** | **Purity** | **RT^a^** | **Obs MW^b^** | **Adduct** | **Purity** |  |
| **1** | 0.94 | 498.5 | [M+H]+ | >95 | 0.93 | 498.2 | [M+H]+ | >95 | >95 |
| **2** | 0.97 | 405.2 | [M+H]+ | >95 | 0.98 | 405.6 | [M+H]+ | >95 | >95 |
| **5** | 0.88 | 509.3 | [M+H]+ | >95 | 0.74 | 509.3 | [M+H]+ | >95 | >95 |
| **5** | 0.89 | 516.2 | [M+H]+ | >95 | 0.90 | 516.2 | [M+H]+ | >95 | >95 |
| **6** | 0.93 | 534.2 | [M+H]+ | 90-95 | 0.93 | 534.2 | [M+H]+ | 85-90 | 85-90 |
| **7** | 0.96 | 552.2 | [M+H]+ | >95 | 0.97 | 552.2 | [M+H]+ | >95 | >95 |
| **8** | 0.92 | 500.2 | [M+H]+ | >95 | 0.92 | 500.1 | [M+H]+ | >95 | >95 |
| **9** | 0.96 | 418.2 | [M+H]+ | >95 | 0.98 | 418.2 | [M+H]+ | >95 | >95 |
| **10** | 0.92 | 436.2 | [M+H]+ | >95 | 0.92 | 436.3 | [M+H]+ | >95 | >95 |
| **11** | 0.92 | 404.3 | [M+H]+ | >95 | 0.92 | 402.6 | [M-H]- | 90-95 | >95 |
| **12** | 1.00 | 422.1 | [M+H]+ | >95 | 1.00 | 422.1 | [M+H]+ | >95 | >95 |
| **13** | 0.96 | 421.1 | [M+H]+ | >95 | 0.87 | 421.1 | [M+H]+ | >95 | >95 |

^a^ RT; Retention time,  ^b^ Obs MW; Observed molecular weight

**Analytical LC-MS details***.*

LC-MS analyses were performed on a Waters Acquity UPLC system fitted with BEH C18 1.7 µM columns (2.1 × 50 mm) and with UV diode array detection (210–400 nm). Positive and negative mass ion detection was performed using a Waters SQD detector. Analyses were performed with either buffered acidic or basic solvents and gradients as detailed below:

Low pH:

Solvent A – Water + 10 mM ammonium formate + 0.1% formic acid
Solvent B – Acetonitrile + 5% water + 0.1% formic acid

High pH:

Solvent A – Water + 10 mM ammonium hydrogen carbonate + 0.1% ammonia solution
Solvent B – Acetonitrile + 0.1% ammonia solution

Gradient:

| Time | Flow rate  (mL/min) | % Solvent A | % Solvent B |
| --- | --- | --- | --- |
| 0 | 0.6 | 95 | 5 |
| 1.2 | 0.6 | 5 | 95 |
| 1.7 | 0.6 | 5 | 95 |
| 1.8 | 0.6 | 95 | 5 |

**Pharmacokinetics**

All studies were conducted after review by the Animal Welfare and Ethical Review Body at CRUK-MI and in accordance with the University of Manchester Policy on the use of animals in research.  All procedures were performed in accordance with the National Home Office regulations under the Animals (Scientific Procedures) Act 1986 and EU Directive 2010/63/EU.

Pharmacokinetics were studied in 6-8 week old male CD-1 mice following single intravenous or oral administration.  Compounds were formulated as solutions comprising up to 10% DMSO with up to 10% Tween80 in saline at a concentration of approximately 1 mM.  Doses were administered at 1 mg/kg (intravenous) or 5 mg/kg (oral gavage) unless otherwise stated.  Blood samples were collected as dried blood spots (Agilent Bond Elut DMS cards) and assayed following solvent extraction using methanol, through a phospholipid removal plate (Phree phospholipid removal plates, Phenomenex) followed by LC-MS/MS analysis. The time-points for blood collection were as follows: oral (0.5, 1, 3, 5 and 24 h); intravenous (0.1, 0.25, 0.75, 2, 5 and 24 h).  The resulting concentration-time data were analyzed by non-compartmental methods (PK Solver, Excel Add-In).
